# Effects of varenicline on the serum levels of olanzapine in male patients with Schizophrenia: a randomized controlled trial

**DOI:** 10.3389/fpsyt.2023.1142419

**Published:** 2023-05-19

**Authors:** Mengling Deng, Zhi Yang, Yanfei Ni, Lingli Zhu, Jiating Xu, Lifeng Zheng, Bo Zhou

**Affiliations:** ^1^Department of Clinical Psychology, The Third Hospital of Quzhou, Quzhou, China; ^2^Department of Psychiatry, The Third Hospital of Quzhou, Quzhou, China

**Keywords:** Schizophrenia, smoking cessation, varenicline, olanzapine, randomized controlled trial

## Abstract

**Background:**

Smoking in patients with Schizophrenia is more common than in the general population. Varenicline, a partial agonist at α4β2 nicotinic acetylcholine receptors, is an effective smoking cessation pharmacotherapy in patients with Schizophrenia. However, its effects on the serum levels of antipsychotics in Schizophrenia are understudied. This study investigated the impact of smoking cessation with varenicline on the serum concentration of olanzapine in patients with Schizophrenia.

**Methods:**

Adult smokers with Schizophrenia were enrolled in a 12-week course of varenicline and placebo for smoking cessation. The serum concentration of olanzapine was measured at baseline and weeks 1, 2, 4, 8, and 12. Data were analyzed with the generalized additive mixed model.

**Results:**

During the 12-week study, the results indicated that olanzapine concentrations increased nonlinearly in the varenicline and placebo groups. Threshold effect analysis suggested that the olanzapine concentrations increased over time until the turning point (week 4). However, there was no significant difference between the two treatment groups.

**Conclusion:**

Varenicline showed safety and efficacy in smoking cessation in people with Schizophrenia.

## Introduction

Globally, cigarette smoking is the second-leading cause of death and the leading cause of disability-adjusted life years (DALYs) (1), predisposing smokers to a wide variety of diseases, including cancer, coronary heart disease, and strokes (2–4). Studies have shown that the estimated rate of smoking prevalence among individuals with Schizophrenia is from 64 to 79% (5–7). In individuals with Schizophrenia, the percentage of severe tobacco dependence is close to 60% (8), especially in male patients, and the average number of cigarettes smoked daily is higher than in the general population (5). It was indicated that heavy smoking was related to elevated substance abuse, increased positive symptom severity, and more frequent psychiatric hospitalization (9). Moreover, the standardized mortality ratio in adults with Schizophrenia is estimated to be more than 3.5 times that in adults in the general population, mainly owing to diseases that are causally related to smoking (10). The excess of deaths due to smoking highlights the critical importance of quitting smoking and treatment of tobacco dependence in settings treating patients with Schizophrenia. Smoking cessation in those with Schizophrenia has been reported to drive a reduced 10-year risk for cardiovascular morbidity and mortality despite abstinence-associated weight gain (11). Existing smoking cessation options for smokers with Schizophrenia consist of cognitive behavioral therapy and three first-line pharmacologic treatments that include Varenicline, bupropion and nicotine replacement therapy either alone or in combination (12, 13).

Varenicline, a partial agonist of the a4ß2 acetylcholine receptor and a full alpha 7 nicotinic acetylcholine receptor agonist (nAChR), is one of the current pharmacotherapies approved by the Food and Drug Administration (FDA) for smoking cessation. High-quality evidence has indicated that varenicline is more efficacious for smoking cessation than placebo, bupropion, or nicotine replacement therapy (14–17). In addition, varenicline has been shown to reduce abstinence-induced withdrawal symptoms, explicitly decreasing cravings. It is a positive reinforcement for smoking cessation in participants with and without schizophrenia (18). Consistent clinical trial evidence supports the safety and efficacy of varenicline for smoking cessation and maintenance of abstinence in Schizophrenia (19). It was shown that varenicline adjuvant therapy did not exacerbate or worsen neuropsychiatric symptoms of Schizophrenia, including overall positive and negative symptoms, and may have a weak effect on improving depression (20–22).

However, data are scarce concerning the potential consequences of varenicline regarding the concentration of antipsychotics used in subjects with Schizophrenia. Such knowledge is essential because the decreased serum concentrations of antipsychotics may contribute to relapses, and increased concentrations could result in adverse effects. Varenicline is mainly eliminated in urine, and research has found that no clinically meaningful drug interactions were identified between varenicline and several representative drugs clinically used (23). Nevertheless, the product of incomplete combustion of organic matter in tobacco (e.g., polycyclic aromatic hydrocarbons) can induce cytochrome P450 isoenzyme 1A2 (CYP1A2) activity through activation of the aryl hydrocarbon receptor, which increases the metabolism of certain antipsychotics, such as clozapine and olanzapine (24). In contrast, the induction of cytochrome P450 isoenzymes decreases after smoking cessation, and serum levels of clozapine and olanzapine may increase, which increases the risk of psychiatric adverse effects (25). Hence, the concentrations of antipsychotics should be monitored closely during smoking cessation. Varenicline is not metabolized by the liver but acts directly on nicotine receptors, thereby blocking tobacco withdrawal symptoms. Theoretically, varenicline has no additional effect on the metabolism of psychotropic medications. However, some studies reported that pharmacotherapeutic cessation aids with varenicline could induce agitation in many patients (26, 27). We suspect that this adverse effect may be attributed to two aspects. One was the effect of smoking cessation itself on antipsychotic metabolism, but not from varenicline, and the other was regarding the agitation induced by varenicline itself, but not antipsychotic serum level related.

Therefore, this study aimed to investigate the effect of varenicline on the serum concentration of olanzapine in smokers with Schizophrenia treated with olanzapine.

## Materials and methods

### Participants

This study was a randomized, 12-week clinical trial examining the effects of varenicline on serum concentrations of olanzapine. Participants were enrolled between July 2017 and August 2018 from the Third Hospital of Quzhou. Subjects were inpatients aged 18–50 years with diagnoses of Schizophrenia as determined by ICD-10. Patients were nicotine-dependent cigarette smokers who smoked an average of 10 or more cigarettes per day during the past year, with nicotine dependence severity (the Fagerstrom Test of Nicotine Dependence, FTND) > 5. Subjects were clinically stable inpatients with total Positive and Negative Syndrome Scale (PANSS) scores <60 during the previous 3 months. There were no changes in medications for the past 3 months. The serum concentrations of olanzapine were more than 9 ng/mL. Exclusion criteria included the presence of drug contraindications, lifetime history of dementia, neurodegenerative disease, or other organic mental disorder, renal insufficiency with estimated creatinine clearance <40 mL/min, or substance abuse or dependence (other than nicotine) in the preceding 12 months.

### Procedure and treatment

Eligible subjects were randomly assigned to either the varenicline group or the matching placebo group. The average age was 36.8 ± 7.7 years. Both subjects and research staff were blinded to study medication assignments. During the 12 weeks, all participants received olanzapine monotherapy. The dose of olanzapine ranged from 10 to 20 mg daily in all participants. Participants were provided 0.5 mg of varenicline or placebo per day for 3 days, 0.5 mg twice daily for 4 days, and then 1.0 mg twice daily for 11 weeks. All subjects agreed to attempt smoking cessation by reducing 25% of cigarettes every 2 weeks, and the smoking “quit date” occurred at the beginning of week 2 (Day 8) of the study.

Additionally, we offered behavioral support to all participants (28). Upon receipt of the recruitment, researchers contact participants to schedule a face-to-face individual informal meeting, which aims to enhance the awareness of the dangers and benefits of smoking cessation, determine a quit date, explain common withdrawal symptoms and advice appropriate methods for relief.

### Assays

The serum concentrations of olanzapine were measured by liquid chromatography-mass spectrometry (LC–MS). Blood samples for assessment of olanzapine were obtained at baseline and at the end of study weeks 1, 2, 4, 8, and 12. The serum was prepared by centrifuging blood samples at 3000 × g for 10 min at room temperature. If not assayed on the same day, the serum was frozen at −80°C until analysis.

### Statistical analysis

We used a generalized additive mixed model (GAMM) approach for repeated measures to estimate the mean difference between the varenicline and placebo groups for each time point. The statistical model included all variables as a fixed effect, except the participant as a random effect. In sensitivity analyses, the model was further adjusted for baseline covariates.

We further applied a two-piecewise linear regression model to examine the threshold effect of the treatment on olanzapine concentrations using a smoothing function. The threshold level (i.e., turning point) was determined using trial and error, including selecting turning points along a predefined interval and then choosing the turning point that gave the maximum model likelihood (29).

All estimates of the treatment effect were calculated with a 95% confidence interval. *p* values less than 0.05 were considered statistically significant. All analyses were performed using Empower (R) (www.empowerstats.com, X&Y solutions, Inc., Boston, MA) and R (http://www.R-project.org).

## Results

### Trial participants

**Figure 1:**
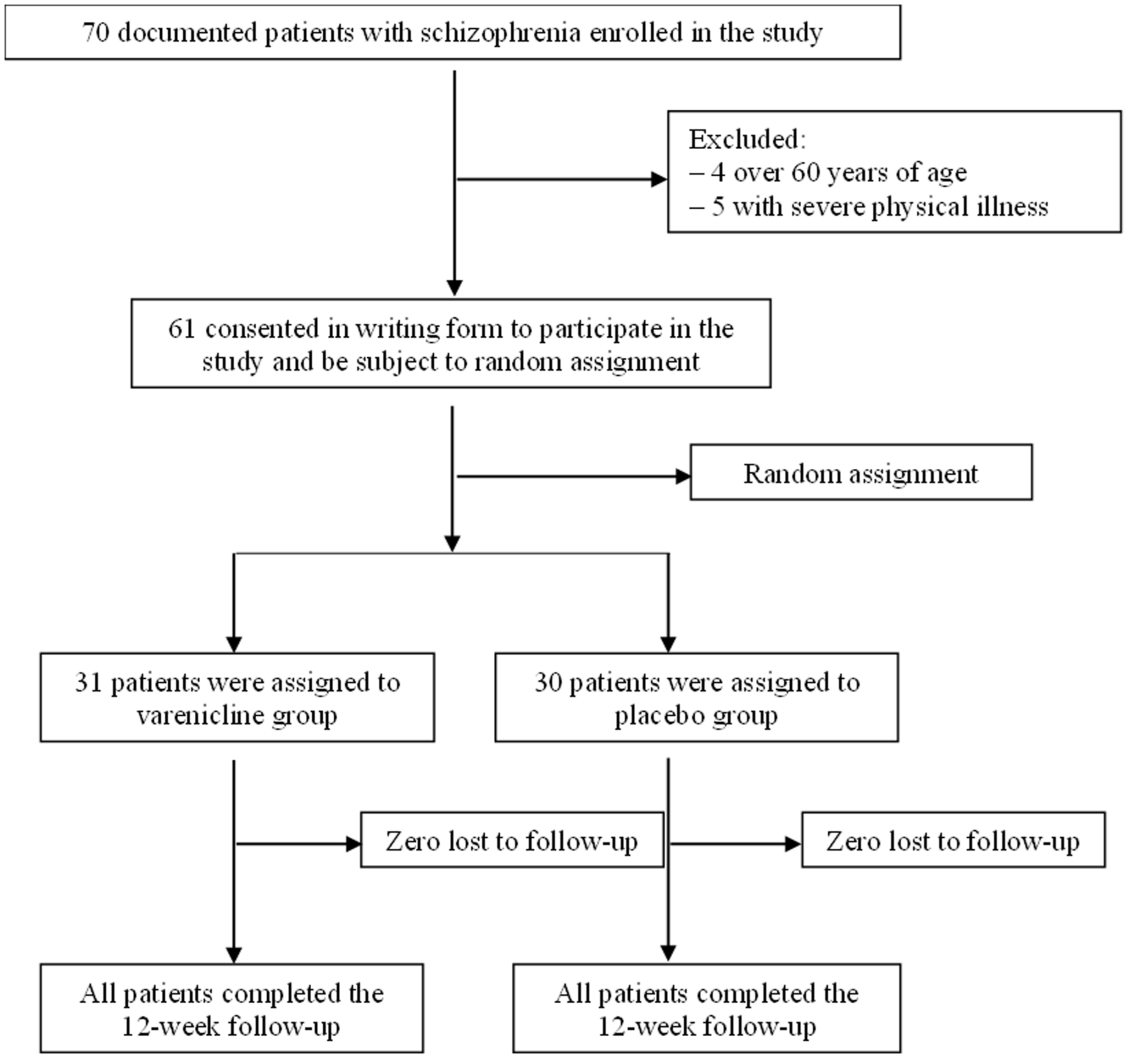
Flowchart of participants through the study.

A total of 70 clients of stop-smoking services were screened, and 61 male patients underwent randomization (30 to the varenicline group and 31 to the matching placebo group). All of the randomly assigned participants completed the 12-week follow-up (Figure 1).

### Participant demographic characteristics at baseline

The baseline characteristics of the study are shown in Table 1. Sixty-one male patients completed the study (30 varenicline and 31 placeboes). The 61 patients had a mean (±SD) age of 36.8 ± 7.7 years. There were no significant differences in baseline characteristics between the two study groups.

**Table 1:** Characteristics of the participants at baseline (mean ± SD).

	Overall (*N* = 61)	Varenicline group (*N* = 30)	Placebo group (*N* = 31)	*p*-value
Age (years)	36.77 ± 7.73	36.17 ± 7.43	37.35 ± 8.09	0.553
Height(m)	1.67 ± 0.07	1.67 ± 0.07	1.67 ± 0.06	0.778
Weight(kg)	66.69 ± 8.83	66.50 ± 10.79	66.87 ± 6.57	0.871
BMI (kg/m^2^)	23.83 ± 2.50	23.76 ± 2.74	23.89 ± 2.29	0.849
Education (years)	9.54 ± 3.49	9.47 ± 3.54	9.61 ± 3.50	0.872
Cigarettes per day	20.16 ± 8.75	20.50 ± 8.84	19.84 ± 8.80	0.771
Duration of smoking (years)	16.67 ± 6.60	16.37 ± 6.57	16.97 ± 6.73	0.725
FTND	7.13 ± 1.18	7.16 ± 1.27	7.10 ± 1.09	0.841
Olanzapine	14.67 ± 4.06	14.68 ± 4.07	14.67 ± 4.14	0.99
Olanzapine in plasma (ng/ml)	48.39 ± 13.01	48.50 ± 11.98	48.29 ± 14.14	0.345

BMI, Body Mass Index.

### Effect of treatment combinations on the concentration of olanzapine

**Figure 2:**
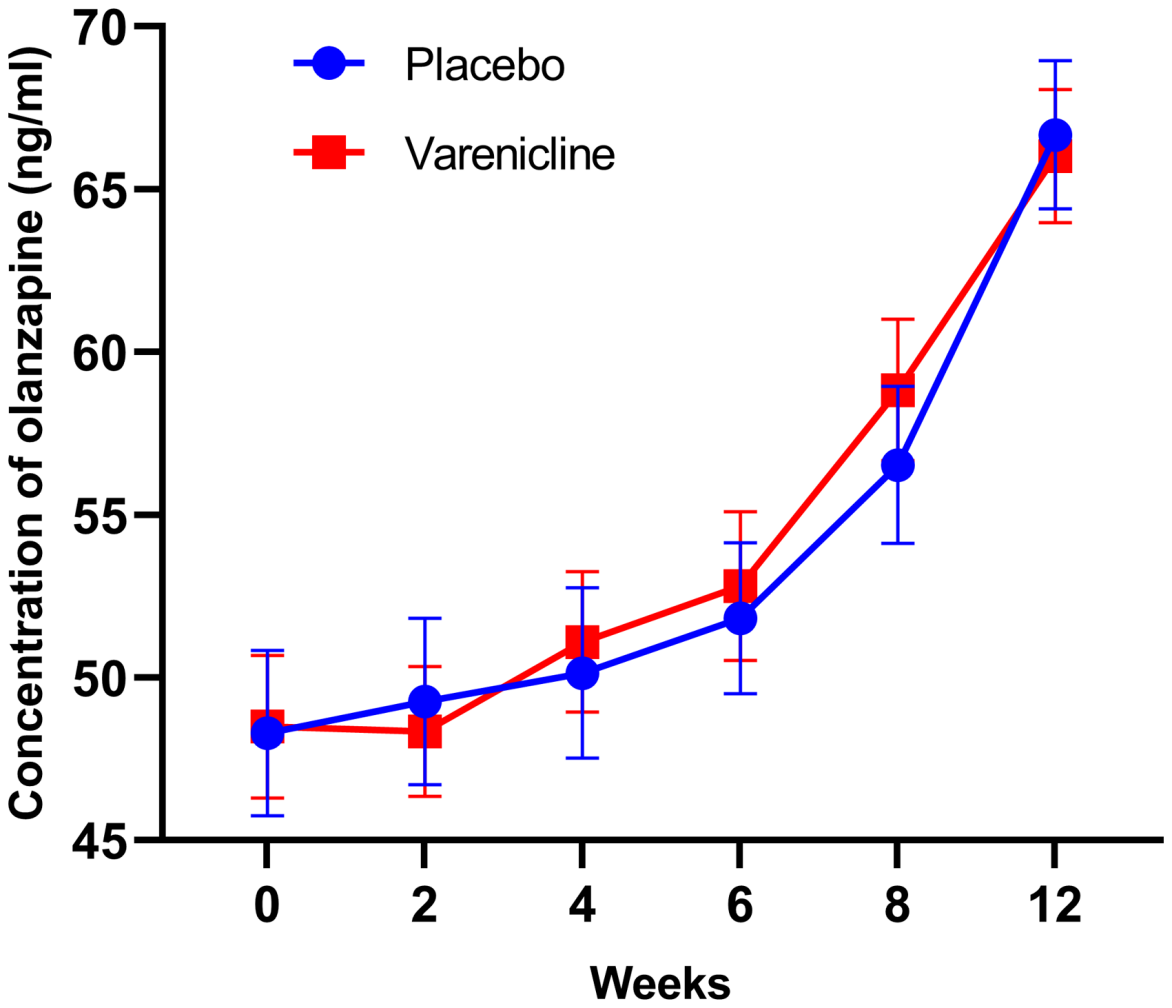
The serum levels of olanzapine from baseline to the 12th weekend in the varenicline and placebo groups in patients with Schizophrenia. The olanzapine concentrations increased over time until the turning point (week 4) both in the two groups.

The olanzapine concentrations at baseline and during the 12-week study duration are indicated in Figure 2. The baseline olanzapine concentrations were 48.5 ± 12.0 ng/mL and 48.3 ± 14.1 ng/mL, which increased to 66.0 ± 11.2 ng/mL and 66.7 ± 12.7 ng/mL for the varenicline group and the placebo group, respectively.

The analysis by the generalized additive mixed model (GAMM) indicated a nonlinear relationship between time and olanzapine concentrations (Figure 2). Threshold effect analysis suggested that the olanzapine concentrations increased over time up to the turning point (week 4, olanzapine concentration = 51.08 ng/mL and week 4, olanzapine concentration = 52.70 ng/mL) for the varenicline group and the placebo group, respectively. Before the end of week 4, the estimated time–response curve was consistent with a horizontal line. Likewise, the time effect on olanzapine concentration increase was not significant (*p* > 0.05). However, the olanzapine concentrations increased until the turning point (week 4); likewise, the correlation coefficients (β) were 1.64 (95% confidence interval [CI]: 0.97 to 2.30; *p* < 0.0001) and 1.83 (95% CI: 1.09 to 2.58; *p* < 0.0001) for the two groups, respectively (Table 2).

**Table 2:** Threshold effect analysis of treatment on olanzapine concentrations using piece-wise linear regression.

	Crude *β* (95% CI) *p*-value	Adjusted *β* (95% CI) *p*-value
*Varenicline*		
<4 weeks	1.14 (−0.19, 2.48) 0.0959	1.14 (−0.12, 2.41) 0.0782
≥4 weeks	1.64 (0.94, 2.34) <0.0001	1.64 (0.97, 2.30) <0.0001
*Placebo*		
<4 weeks	0.64 (−0.90, 2.17) 0.4180	0.64 (−0.78, 2.05) 0.3797
≥4 weeks	1.83 (1.02, 2.64) <0.0001	1.83 (1.09, 2.58) <0.0001

Crude: no adjustment.

Adjusted: adjusted for the baseline age, height, weight, BMI, education, cigarettes per day, and duration of smoking years.

The unadjusted increase in olanzapine concentrations was 3.53 ng mL per week (95% CI, 1.07 to 6.00; *p* = 0.005) in the group that received placebo and 4.31 ng/mL per week in the group that received varenicline (95% CI, 1.85 to 6.78; p = 0.005) at the end of week 4 (Table 3) and a more significant increase at the end of week 12 (18.38 ng/mL per week, 95% CI, 15.91 to 20.85; *p* < 0.001) in the group that received placebo and the group that received varenicline (17.53 ng/mL per week, 95% CI, 15.06 to 20.00; *p* < 0.001).

**Table 3:** Generalized additive mixed model–main effects in changes from baseline.

	Weeks 4		Weeks 12	
	Time *β* (95%CI)	Group *β*(95% CI)	Time *β* (95% CI)	Group *β* (95% CI)
*Crude*				
Varenicline	4.31(1.85, 6.78)	0.78(−2.73, 4.30)	17.53(15.06, 20.00)	−0.85 (−4.37, 2.66)
Placebo	3.53 (1.07, 6.00)	Reference	18.38 (15.91, 20.85)	Reference
*Adjusted*				
Varenicline	4.31(1.82, 6.80)	0.78 (−2.77, 4.34)	17.53 (15.04, 20.02)	−0.85 (−4.37, 2.66)
Placebo	3.53 (1.04, 6.02)	Reference	18.38 (15.89, 20.87)	Reference

Crude: no adjustment.

Adjusted: adjusted for the baseline age, height, weight, BMI, education, cigarettes per day, and duration of smoking years.

An analysis of the course of olanzapine concentrations using the mixed procedure indicated that there was no statistically significant difference between the two treatment groups at the end of week 4 (β = 0.78, 95% CI, −2.73 to 4.30; *p* = 0.662) and the same pattern was observed at the other time points (Table 3).

After adjusting for the baseline age, height, weight, BMI, education, cigarettes per day, and duration of smoking years, the estimates of olanzapine concentrations also showed no significant difference between the two groups at the end of week 4 (β = 0.78, 95% CI, −2.77 to 4.34; *p* = 0.666) and the same pattern was observed at the other time points (Table 3).

## Discussion

In this randomized controlled study, we investigated the effects of a 12-week smoking cessation treatment with varenicline plus behavioral intervention or matching placebo plus behavioral intervention on olanzapine concentration in male patients with Schizophrenia. The results from this study indicate that smoking cessation was associated with a higher serum olanzapine concentration. We further revealed a threshold effect based on the serum olanzapine concentration. However, this trial did not show a significant difference in olanzapine concentration between the varenicline and placebo groups in male patients with Schizophrenia. This is the first clinical study to provide clear evidence of a nonlinear association of olanzapine concentration changes over time in male smoking-cessation patients with Schizophrenia.

CYP1A2 is a member of the CYPs, which is associated with the metabolism of several drugs (30). Meanwhile, the enzyme induction of CYPs was implicated in several factors, such as gender, race, genetic polymorphisms, and exposure to inducers. Tobacco smoking is known to be responsible for the induction of cytochrome P450 (30). Tobacco smoking is known to be responsible for the induction of c CYP1A2 (31). The atypical antipsychotic olanzapine is metabolized by CYP1A2 and CYP-2D6 (32). Compared with nonsmokers, the clearance of olanzapine increased by 98% in smokers (33). Our study found that serum olanzapine concentration increased significantly at the end of week 4 with smoking cessation treatment, consistent with a previous report (20).

Varenicline is a partial agonist of α4β2 nAChRs linked to nicotine’s reinforcing effects and maintenance of smoking behavior. Therefore, varenicline is considered to increase the incidence of psychiatric adverse events because dopamine is hypothesized to be involved in developing and continuing various psychiatric illnesses, including schizophrenia and mood disorders (34–36). On the other hand, accumulating evidence suggests that a7 nAChRs play a role in the pathogenesis of Schizophrenia (37, 38). However, the evidence indicates that varenicline did not significantly increase neuropsychiatric adverse events relative to placebo (39). The results of our study did not show a significant difference in olanzapine concentration attributable to varenicline relative to the placebo. Our findings showed for the first time that the safety of varenicline, in terms of the olanzapine concentration βs values, was similar for the two treatment groups. Accordingly, it was speculated smoking cessation itself effected antipsychotic metabolism, but not varenicline, and the psychiatric adverse events, such as agitation, was induced by varenicline itself, but not olanzapine serum level, which was needed to be further investigated.

### Limitations

The present study has several limitations. First, we included smokers with Schizophrenia who were stable and treated with olanzapine alone. Thus, these selection effects might have influenced the results of our study, and our findings might not generalize to those who are symptomatically unstable or treated with two or more antipsychotics. Second, this study is short (12 weeks) and therefore does not reflect the effect of long-term smoking cessation in male patients suffering from Schizophrenia. And the frequent measures might not reflect a real-world smoking cessation process. Third, we enrolled patients who smoked, on average, at least 10 cigarettes per day and who were moderately nicotine dependent. Thus, our findings might not generalize to lighter, less severely dependent smokers. Fourth, the participants were almost all men. Therefore, this study did not reflect sex differences. However, the percentage of women smoking in China is relatively small. Thus, to some extent, this study mirrored real-world conditions.

## Conclusion

In conclusion, our study showed that the serum concentration of olanzapine increased significantly after 4 weeks of smoking cessation treatment. Varenicline, as a smoking cessation aid in Schizophrenia, did not impact the olanzapine concentration. These results provide further evidence that varenicline with behavioral intervention can be used safely by psychiatrically stable schizophrenia smokers. We also suggest monitoring the serum levels of olanzapine in addition to a thorough clinical follow-up when using smoking cessation pharmacotherapy in male patients with Schizophrenia.

## Data availability statement

The raw data supporting the conclusions of this article will be made available by the authors, without undue reservation.

## Ethics statement

The studies involving human participants were reviewed and approved by the Ethics Committee of the Third Hospital of Quzhou. The patients/participants provided their written informed consent to participate in this study.

## Author contributions

MD and BZ designed the study. MD, ZY, YN, LinZ, and JX collected the samples and clinical information. MD, LifZ, and BZ analyzed and discussed the experimental result. MD wrote the first draft of the manuscript. All authors contributed to the article and approved the final manuscript.

## Funding

This work supported by the Medical and Health Science and Technology Project of Zhejiang Province (Grant Number: 2018KY879) and the Science and Technology Plan Project of Quzhou (Grant Number: 2022 K83).

## Conflict of interest

The authors declare that the research was conducted in the absence of any commercial or financial relationships that could be construed as a potential conflict of interest.

## Publisher’s note

All claims expressed in this article are solely those of the authors and do not necessarily represent those of their affiliated organizations, or those of the publisher, the editors and the reviewers. Any product that may be evaluated in this article, or claim that may be made by its manufacturer, is not guaranteed or endorsed by the publisher.
